# Modelling the impact of correlations between condom use and sexual contact pattern on the dynamics of sexually transmitted infections

**DOI:** 10.1186/s12976-018-0078-9

**Published:** 2018-05-31

**Authors:** Nao Yamamoto, Keisuke Ejima, Hiroshi Nishiura

**Affiliations:** 1 Graduate School of Medicine, Hokkaido University, Hokkaido, Japan; 2 CREST, Japan Science and Technology Agency, Saitama, Japan; 3 Institute of Industrial Science, The University of Tokyo, Tokyo, Japan; 4 Epidemiology and Biostatistics, School of Public Health, Indiana University Bloomington, Bloomington, IN USA

**Keywords:** Unprotected sex, Partnership, Condom, HIV, Gonorrhoea, Chlamydia

## Abstract

**Background:**

It is believed that sexually active people, i.e. people having multiple or concurrent sexual partners, are at a high risk of sexually transmitted infections (STI), but they are likely to be more aware of the risk and may exhibit greater fraction of the use of condom. The purpose of the present study is to examine the correlation between condom use and sexual contact pattern and clarify its impact on the transmission dynamics of STIs using a mathematical model.

**Methods:**

The definition of sexual contact pattern can be broad, but we focus on two specific aspects: (i) type of partnership (i.e. steady or casual partnership) and (ii) existence of concurrency (i.e. with single or multiple partners). Systematic review and meta-analysis of published studies are performed, analysing literature that epidemiologically examined the relationship between condom use and sexual contact pattern. Subsequently, we employ an epidemiological model and compute the reproduction number that accounts for with and without concurrency so that the corresponding coverage of condom use and its correlation with existence of concurrency can be explicitly investigated using the mathematical model. Combining the model with parameters estimated from the meta-analysis along with other assumed parameters, the impact of varying the proportion of population with multiple partners on the reproduction number is examined.

**Results:**

Based on systematic review, we show that a greater number of people used condoms during sexual contact with casual partners than with steady partners. Furthermore, people with multiple partners use condoms more frequently than people with a single partner alone. Our mathematical model revealed a positive relationship between the effective reproduction number and the proportion of people with multiple partners. Nevertheless, the association was reversed to be negative by employing a slightly greater value of the relative risk of condom use for people with multiple partners than that empirically estimated.

**Conclusions:**

Depending on the correlation between condom use and the existence of concurrency, association between the proportion of people with multiple partners and the reproduction number can be reversed, suggesting the sexually active population is not necessary a primary target population to encourage condom use (i.e., sexually less active individuals could equivalently be a target in some cases).

## Background

Sexually transmitted infection (STI) remains to be a serious concern of public health, involving more than 30 pathogens [1, 2]. Of these, major eight STIs include syphilis, gonorrhoea, chlamydia, trichomoniasis, hepatitis B virus infection, herpes simplex virus infection, human immunodeficiency virus (HIV) infection, and human papilloma virus infection (HPV), that are mainly linked to sexual contact. Given that one cannot fully rely on treatment of these diseases, it is vital that prevention should be the main stream of interventions. Indeed, even among curable STIs, acute course of infection can sometimes develop to urethritis, cervicitis, genital ulceration and pelvic inflammatory disease (PID) [3]. The PID caused by Chlamydia trachomatis infection can result in multitudes of adverse pregnancy outcomes including miscarriage [4, 5]. Moreover, bacterial STIs including syphilis and chlamydia are known to be associated with elevated risk of HIV infection [6].

Without any doubt, the mainstream of STI prevention is to use the male latex condom. While the condom was originally invented as one of contraceptive options, its prophylactic use has been shown to be useful through the epidemic of HIV/AIDS and protective efficacy of the latex condom have been demonstrated for a variety of STI pathogens [7]. The condom is nowadays in the World Health Organization’s list of essential medicines needed in the social system. While numerous studies took place on the preventive use of condom including its proper use, one of main concerns has been whether a partner at risk actually employs this sheath-shaped barrier device during the potentially unsafe sexual contact.

Who should then wear the condom during sexual contact? Among non-experts, there has been a misconception that only people who have multiple partners should use condom [8]. Considering the frequency and diversity of sexual contact that sexually active individuals experience, wearing condom among people with multiple partners might be a reasonable advice. Nevertheless, it could also be true that people with more sexual partners generally are more aware of the importance of prevention such as utilizing condoms [9], and awareness of those with a steady partner may not be as high as those with casual partners. No systematic review of prospective studies has taken place to examine the relationship between condom use and sexual contact pattern, such as type of partnership or the existence of concurrency.

The purpose of the present study is to examine the correlation between condom use and sexual contact pattern and clarify its impact on the transmission dynamics of STIs using a mathematical model. Our task is twofold. First, we examine whether sexual contact pattern is associated with frequency of condom use through systematic review and meta-analysis. The definition of contact pattern can be broad, but we focus on two specific aspects: (i) type of partnership (steady or casual) and (ii) existence of concurrency (single or multiple partners). We have to note that the type of partnership characterizes the relationship of a particular couple experiencing a sexual contact, whereas the existence of concurrency purely dictates the number of sexual contact during the same time period. For example, one can have two concurrent partners, one partnership is steady and the other is causal. Second, if the number of contact is associated with condom use, we investigate which (single vs. multiple) is more likely to contribute to the transmission at a population level, employing a mathematical model that captures the transmission dynamics.

## Methods

The present study is composed of two major analytic steps, i.e. (i) a systematic review of literature and (ii) a mathematical modelling of the transmission dynamics.

### Search strategy

Studies containing data on the correlation between condom use and sexual contact pattern were retrieved from MEDLINE (PubMed) and Web of Science electronic databases on 24 April 2016. We used the following free text search terms in “All fields”:

- #1: “condom use” OR “safer sex” OR “unprotected intercourse” OR “unprotected sex” OR “unsafe sex”
- #2: partner OR partners OR partnership
- #3: prospective OR cohort
- #4: gay[TI] OR homosexual[TI] OR homosexuals[TI] OR lesbian[TI] OR “men who have sex with men”[TI] OR MSM[TI]
- #5: “injecting drug user” OR “injecting drug users” OR “injection drug use” OR “injection drug users” OR IDU
- #6: #1 AND #2 AND #3 NOT #4 NOT #5

We have limited research studies to only those designed as prospective or cohort studies, because relative risk estimate has been sought to calculate the excess risk of concurrent partnership for not using condom.

### Study selection

The systematic search of literature was conducted from July 2014 to April 2016. All titles identified by the search strategy were independently screened by two authors (N.Y. and K.E.). Abstracts of potentially relevant titles were then reviewed for eligibility, and appropriate articles were selected for closer examination if any description of correlation between condom use and sexual contact pattern was given.

### Systematic review

Although sexual contact pattern can be broadly interpreted and defined in various ways, we focus on two specific aspects as explanatory variables: (i) whether the partnerships were steady or casual and (ii) whether the persons had a single or multiple (concurrent) partners at the same time. Our dichotomous outcome variable is the use of condom. The risk of using condom was calculated as the proportion of identified condom use partnership (or persons) divided by the total number of a particular type of partnership (or persons). Then, the relative risk of using condom given a type of partnership (or person) was estimated for each extracted study, followed by meta-analysis. Statistical heterogeneity was assessed by Cochran’s Q and I^2^ statistic which represent the extent of the degree of variation between studies [10, 11]. All statistical data were analysed using a statistical software R version 3.1.2 (R Core Team, Vienna, Austria, 2017) and the library ‘metafor’ was used for forest plot.

### Epidemic model with populations with or without concurrency (with a single or multiple partners)

Using the estimated association between condom use and sexual contact pattern, we examine its role in determining the transmission dynamics of STI. Considering the heterogeneous sexual contact pattern, both the number of sexual partners and the types of partnership (i.e., steady or casual) would play key roles in modulating the epidemic dynamics. However, hereafter, we focus on modelling concurrency. The population is divided into four groups due to two sex (i.e., male and female) and two different categories with a single or multiple partners. The epidemic dynamics of STI is described by the following next generation matrix (NGM), ***K***:

1 $$ \boldsymbol{K}\sim \boldsymbol{C}=\left[\begin{array}{cccc}& & {c}_{m_1{f}_1}& {c}_{m_1{f}_2}\\ {}& 0& {c}_{m_2{f}_1}& {c}_{m_2{f}_2}\\ {}{c}_{f_1{m}_1}& {c}_{f_1{m}_2}& 0& \\ {}{c}_{f_2{m}_1}& {c}_{f_2{m}_2}& & \end{array}\right] $$

where ***C*** stands for the contact matrix, composed of the contact rate per unit time within and between different groups of people. That is, the element *c*_ij_ represents the rate of sexual contact that one individual in group *j* experiences with partner(s) in the group *i*. Subscripts *m*_i_ and *f*_i_ represents male and female groups with a single or multiple sexual partners *i*, i.e., *i* = 1 representing population with multiple partners and 2 with a single partner. For simplicity, we ignore the issue of homosexual transmission in this model. Assuming that there would be no biological difference among groups with respect to infectiousness, susceptibility and the incubation period, the next generation matrix, ***K*** is assumed as proportional to the contact matrix, ***C***. The eigenvalue of ***K*** would yield the basic reproduction number, *R*_0_, i.e., the average number of secondary cases generated by a single typical infectious individual in a fully susceptible population, which acts as the threshold of observing a major epidemic. To compute ***K***, each element of the contact matrix is parameterized as follows:

2 $$ {c}_{m_1{f}_1}= wc\left\{\theta +\left(1-\theta \right)\frac{pw}{pw+\left(1-p\right)}\right\}, $$

3 $$ {c}_{m_2{f}_1}= wc\left(1-\theta \right)\frac{1-p}{pw+\left(1-p\right)}, $$

4 $$ {c}_{m_1{f}_2}=c\left(1-\theta \right)\frac{pw}{pw+\left(1-p\right)}, $$

5 $$ {c}_{m_2{f}_2}=c\left\{\theta +\left(1-\theta \right)\frac{1-p}{pw+\left(1-p\right)}\right\}, $$

6 $$ {c}_{f_1{m}_1}= wc\left\{\theta +\left(1-\theta \right)\frac{pw}{pw+\left(1-p\right)}\right\}, $$

7 $$ {c}_{f_2{m}_1}= wc\left(1-\theta \right)\frac{1-p}{pw+\left(1-p\right)}, $$

8 $$ {c}_{f_1{m}_2}=c\left(1-\theta \right)\frac{pw}{pw+\left(1-p\right)}, $$

9 $$ {c}_{f_2{m}_2}=c\left\{\theta +\left(1-\theta \right)\frac{1-p}{pw+\left(1-p\right)}\right\}, $$

where *c* is the contact rate of people with a single partner only, and *w* scales the relative contact rate of people with multiple partners as compared with single partner only. The parameter *θ* is referred to as the assortativity coefficient, which describes the proportion of contacts that are spent within the same group [12]. Thus, (1-*θ*) of the contacts are spent randomly, or in the above formulation, that is equivalent to say proportional to relative population size of each group to be used as the weight. In the extreme case, *θ* = 0 and 1 corresponds to the random mixing and fully assortative mixing (i.e. contacts occur only in the same groups), respectively. For example, sexual contact rate of a female with multiple partners (*f*_1_) with a male with multiple partners (*m*_1_), ${C}_{m_1{f}_1}$, is described as $wc\left\{\theta +\left(1-\theta \right)\frac{pw}{pw+\left(1-p\right)}\right\}$, because, among the total of contact, *wc*, the proportion *θ* is distributed to the contacts with male with multiple partners, and the rest of total contact rate, (1-*θ*) is randomly distributed to the contacts with male with or without concurrency. *p* is the proportion of people with multiple partners among the entire population, and the complement (1-*p*) describes the proportion of people with a single partner only.

**Fig. 1:**
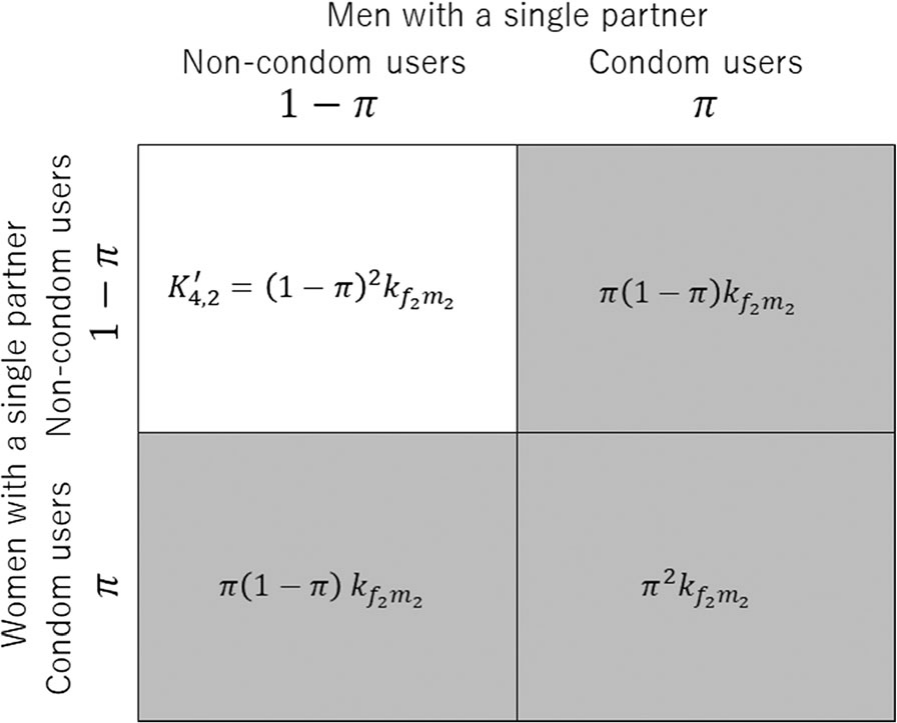
Example of the construction of next generation matrix. Based on the next generation matrix without any intervention, ***K***, next generation matrix with condo use, ***K****′* was constructed. As an example, the illustration of ${K}_{4,2}^{\hbox{'}}\left(={K}_{f_2,{m}_2}^{\hbox{'}}\right)$ is shown. This element describes the transmission from men with a single partner to women with a single partner

Adding onto the abovementioned model, we account for the condom use. Assuming that condoms can perfectly prevent infection, the proportion of condom users depends only on the existence of concurrency (i.e. with a single or multiple partners). Not only male, but hereafter we consider that an identical impact is seen in female as well. The NGM under intervention, ***K***′ is described as

10 $$ {K}^{\hbox{'}}=\left[\begin{array}{cccc}& & {\left(1- q\pi \right)}^2{k}_{m_1{f}_1}& \left(1- q\pi \right)\left(1-\pi \right){k}_{m_1{f}_2}\\ {}& 0& \left(1- q\pi \right)\left(1-\pi \right){k}_{m_2{f}_1}& {\left(1-\pi \right)}^2{k}_{m_2{f}_2}\\ {}{\left(1- q\pi \right)}^2{k}_{f_1{m}_1}& \left(1- q\pi \right)\left(1-\pi \right){k}_{f_1{m}_2}& & \\ {}\left(1- q\pi \right)\left(1-\pi \right){k}_{f_2{m}_1}& {\left(1-\pi \right)}^2{k}_{f_2{m}_2}& 0& \end{array}\kern1em \right], $$

where *k*_ij_ represents the (*i*,*j*)-th element of the next generation matrix ***K*** in the absence of intervention, and *π* represents the coverage of condom use among people with multiple partners. *q* is the relative coverage of condom use among the people with a single partner alone. In each element with *q* and *π*, *π* appears twice, because condom use can perfectly prevent infection, risky sexual intercourse happens only between non-condom males and non-condom use females (Fig. 1). As an example, the illustration of ${K}_{4,2}^{\hbox{'}}\left({K}_{f_2,{m}_2}^{\hbox{'}}\right)$ is shown in Fig. 1. This element describes the transmission from men with a single partner to women with a single partner. The proportion of population with condom use is *π* for each population, contacts involving at least one condom user (grey shaded parts) are excluded from the transmission dynamics. Thus the element is composed of only the transmission between non-condom users. We can compute the largest eigenvalue of ***K***′ that yields the effective reproduction number.

### Scenario analysis

To investigate the relationship between epidemiological dynamics and concurrency, we examined the sensitivity of the effective reproduction number to proportion of people with multiple sexual partners (*p*). The set of assumed parameters is shown in the Table 1.

**Table 1:** Parameters for sensitivity analysis of the sexual transmitted infection

Parameters	Description	Assumed values
*R*_0_	Basic reproduction number	3.65 [33]
*c*	Rate of sexual contact among people with steady (or single) partner only	Back-calculated from *R*_0_
*w*	Relative frequency of sexual contact among people with casual (or multiple) partners	4.0 (Assumed)
*π*	Coverage of condom use	0.49 (Estimated in systematic review)
*q*	Relative coverage of condom use among people with multiple partners	1.32 (Estimated in systematic review)
*p*	Proportion of people with multiple partners	0.30 (Assumed)
*θ*	Assortativity coefficient (i.e., proportion of contacts that are spent for within group mixing)	0.20 (Assumed)

### Ethical considerations

The present study analysed only published articles and handled openly available data. As such, the present study did not require ethical approval.

## Results

### Systematic review

**Fig. 2:**
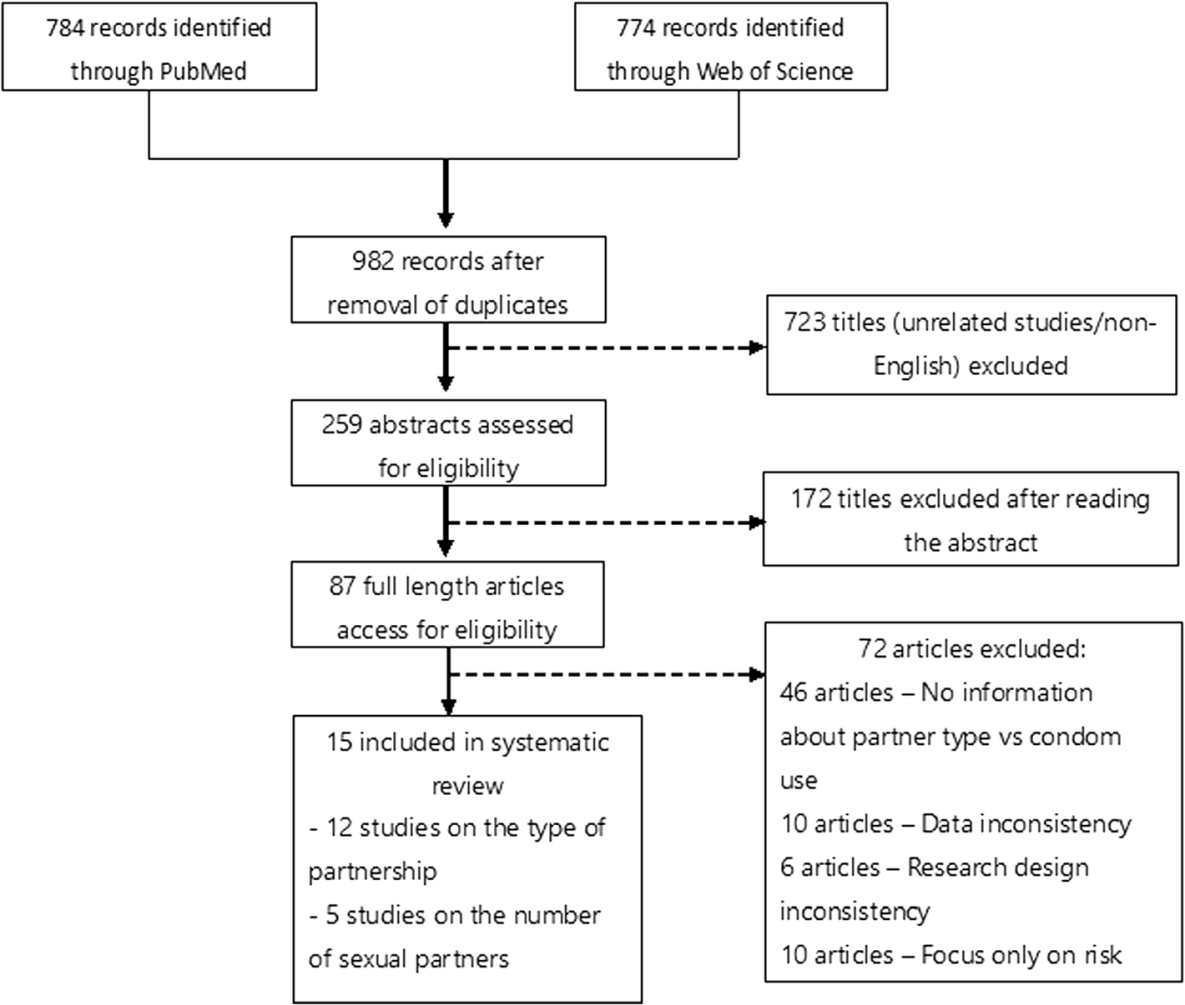
Flow diagram of study selection. Among a total of 784 and 774 records identified by using MEDLINE and Web of Science, respectively, a total of 15 studies fulfilled inclusion criteria and were included in our systematic review

We retrieved 1558 potential publications based on two different databases (Fig. 2), of which 259 were considered potentially eligible for assessing the abstract. Of 1299 excluded studies, 576 appeared to be duplicates (i.e. hit on both databases), 9 were not in English, and 714 were determined to be irrelevant subject. Reading abstracts, 172 titles were excluded because they were regarded as irrelevant to our research subject. Reviewing the full text, another 70 studies were excluded as 46 did not include information regarding correlation between condom use and partnership, and it was hard to extract the data from 10 studies, the research design did not meet our criteria in 6 articles, and 10 articles only focused on particular risk groups such as commercial sex workers or intravenous drug users. Finally, 15 studies were determined to be eligible and included in this systematic review [9, 13–26]. Of the included 15 studies, a total of 12 studies described the association between condom use and having steady partner (or casual partner(s)). Of the 15 studies, 5 studies described the association between condom use and concurrency (i.e. single partner alone or multiple partners).

**Fig. 3:**
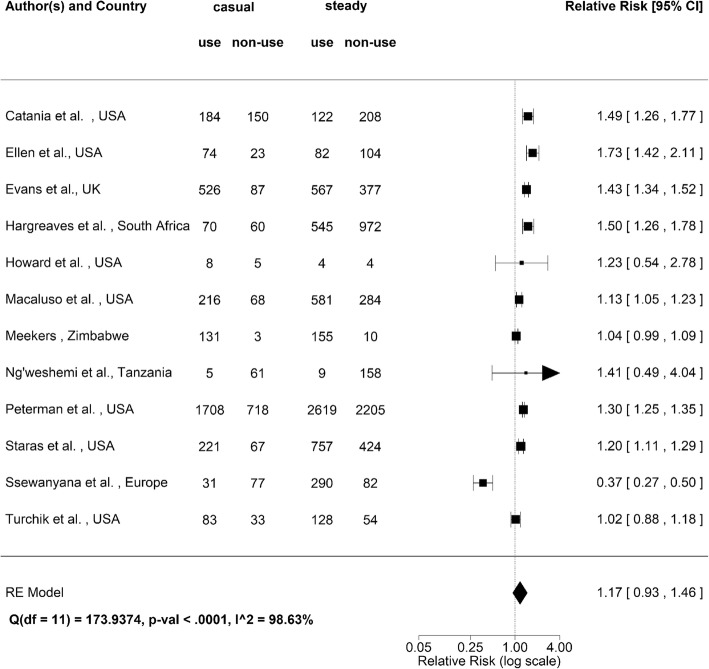
Forest plot of potential association between condom use and partner type (i.e., steady or casual). Centre of each square points the relative risk with its size reflecting the sample size. Whiskers extend to lower and upper 95% confidence intervals. The right arrow in the Tanzania study indicates that the upper bound is greater than upper limit of our horizontal axis scale. Diamond represents the group estimate based on random effects model. I^2^ statistic shows the extent of heterogeneity

Figure 3 summarizes the characteristics of the selected 12 different studies on the type of partnerships. There was variation in literary expression to define the type of partnership. We define the use of following words as the signature of steady partner: (i) boyfriend/girlfriend, (ii) main, (iii) relationship partner, (iv) regular and (v) primary. Conversely, we define the use of following terms as casual: (i) one-time, (ii) non-regular, (iii) secondary and (iv) short-term. With respect to the use of condom, its use was quantified in different manner by different studies. In five of the included studies, data were collected and classified as non-yes-or-no responses in a categorical manner such as “never”, “occasionally/often” or “always”. Because seven studies show only dichotomized data with yes or no responses, categories indicating any degree of condom use (e.g. “sometimes” or “always”) are presented as yes as opposed to non-existent condom use (e.g. “never” or “none”) with the results presented as “condom use” or “condom non-use” to ensure the consistency.

The relative risk of using condom given casual partnership was significantly greater than the value of 1 in 7 published studies. Only 1 study in Europe indicated that those with casual partners less frequently used condom. Weighted mean of the relative risk based on random effects model was estimated at 1.2 (95% confidence interval (CI): 0.9, 1.5). Heterogeneity was identified to be high with I^2^ value estimated at 98.6%.

**Fig. 4:**
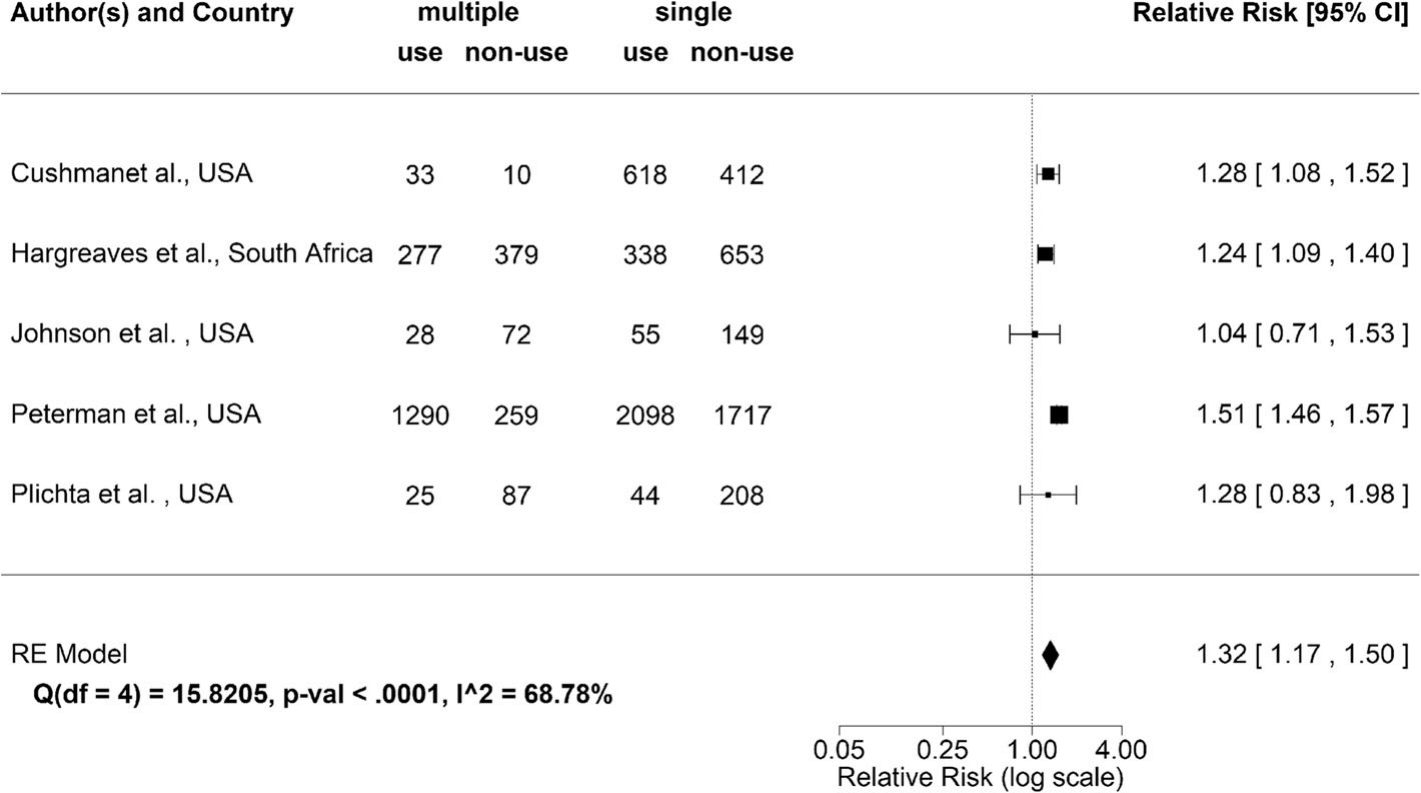
Forest plot of potential association between condom use and concurrency (i.e., having 1 partner alone or 2 or more concurrent partners). Centre of each square points the relative risk with its size reflecting the sample size. Whiskers extend to lower and upper 95% confidence intervals. Diamond represents the group estimate based on random effects model. I^2^ statistic shows the extent of heterogeneity

Figure 4 summarizes the characteristics of the selected 5 studies on concurrency. The relative risk of using condom given multiple partners was significantly greater than the value of 1 in 3 published studies. None of the included studies indicated that those with multiple partners less frequently used condom. Weighted mean of the relative risk based on random effects model was estimated at 1.3 (95% confidence interval (CI): 1.2, 1.5). If a fixed effects model was employed, the weighted mean was estimated at 1.5 (detailed Results not shown). Heterogeneity was again identified to be high with I^2^ value estimated at 68.8% from random effects model.

**Fig. 5:**
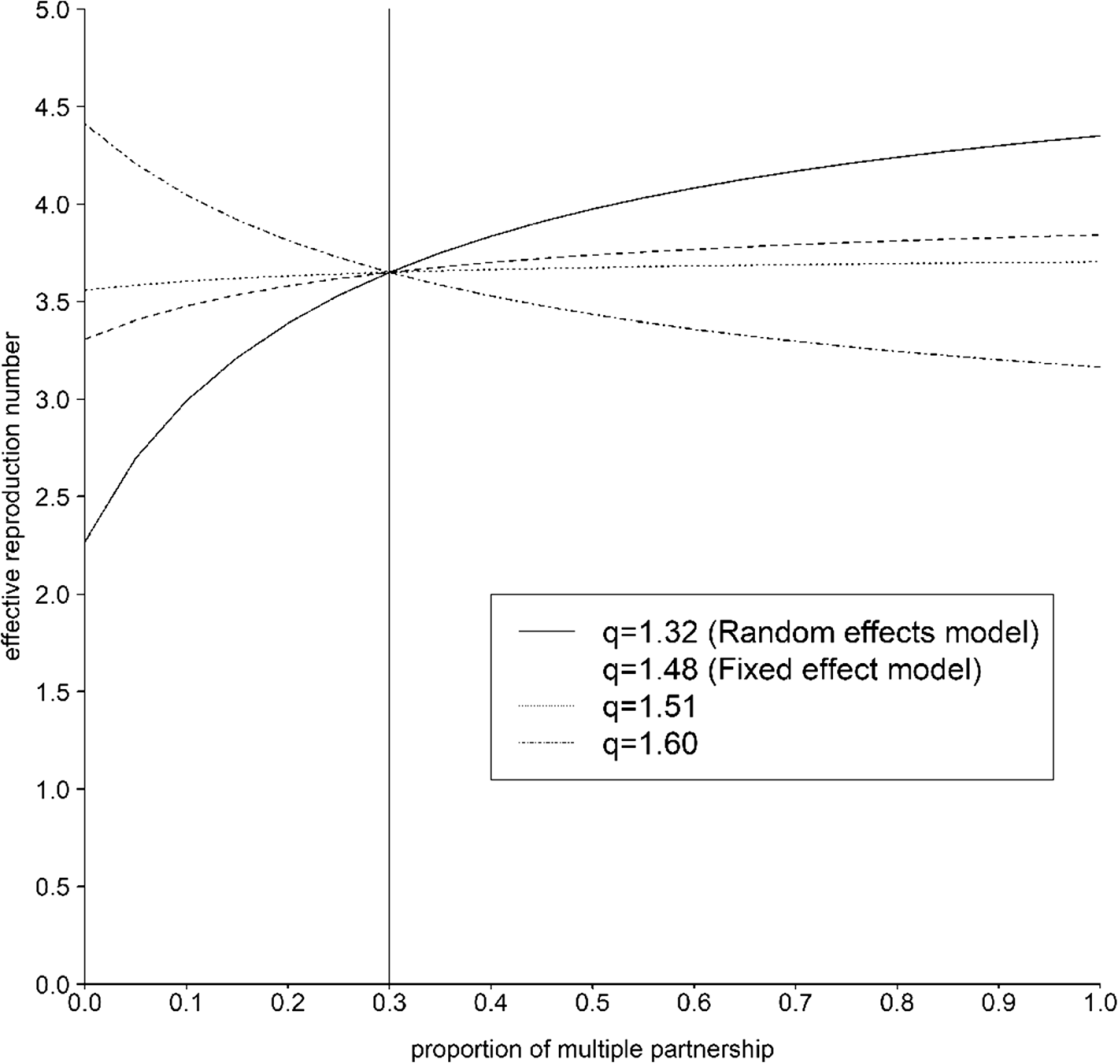
Sensitivity of the transmissibility to the fraction of people with multiple sexual partners. Different lines represent the estimate of transmissibility (effective reproduction number) with different values of *q*, the relative risk of condom use due to more active sexual partnership. The value of 1.32, 1.48 and 1.51 were derived from systematic review and 1.60 is what the authors assumed

### Epidemiological modelling

Based on the relative risk of condom use given multiple partners, the effect size was estimated at 1.32 or 1.48 using random effects or fixed effect models, respectively. Among included studies, the greatest effect size by published study was 1.51. In addition to these values, we examined another bigger effect size at 1.60. Consequently, we examined the sensitivity of effective reproduction number to the proportion of the multiple partnership in the population using the fixed values of *q* at 1.32, 1.48, 1.51 and 1.60 (Fig. 5). Using the published realistic values of *q* (i.e., 1.32), the effective reproduction number increased if the proportion of people with multiple partners (*p*) was elevated. This was also the case for *q* = 1.48 and 1.51, but the extent of increase was almost diminished.

However, if *q* is as high as 1.60, the relationship between the effective reproduction number and proportion of people with multiple partners was reversed, i.e., as people become more likely to have multiple partners, the reproduction number takes smaller value. That is, depending on parameters that govern the sexual transmission dynamics (especially, the relative risk *q* and perhaps also the relative contact frequency *w* and assortativity *θ*), the resulting positivity of the relationship between the reproduction number and the proportion of people with multiple partners is determined.

## Discussion

The present study analysed the correlation between condom use and type of partnership or concurrency by conducting systematic review of published literature. Subsequently, the impact of the correlation between condom use and concurrency on the transmission dynamics was examined computing the effective reproduction number and using empirically estimated relative risk of condom use among people with multiple partners. Empirical datasets indicated that a greater number of people used condoms during sexual contact outside of an ongoing relationship (casual contact) than with a steady partner. Furthermore, people with multiple partners use condoms more frequently than people with a single partner alone. Embedding the empirical estimate onto the mathematical model, a positive relationship between the reproduction number and the proportion of people with multiple partners was identified. Nevertheless, the relationship was reversed to be negative by employing a greater value of the relative risk of condom use given multiple partners than that empirically estimated.

In foregoing studies of STI modelling, there has been a trend to focus on partnership via risk-based modelling approach incorporating contact frequency to constitute host type [27] and also employing the so-called pair formation modelling approaches [28–31]. There are several studies in which sexual behaviour and condom use was modelled and their association with disease spread dynamics was examined. Most of those published studies treated sexual behaviour and condom use independently. Azizi et al. [32] is similar to ours as they incorporated correlation between condom use and heterogeneous risk behaviour. In contrast, we focused only on two specific aspects of sexual behaviour, i.e., type of partnership and concurrency. Especially in the modelling part, we modelled concurrency considering differential contact rate (*c* vs *cw*). We did not (or could not) incorporate all aspects of sexual behaviour into simplistic model, but our formulation has made each component of the model (i.e. parameters) interpretable and observable. For example, we can count the number of sexual contacts, which is modelled as *c* or *cw*, population with multiple partners can be easily identified, although this might be self-reported. This is important especially when the results are translated into public health practice or when parameters are estimated for different populations.

In case the relative risk *q* (i.e., relative condom coverage for people with multiple partners) is in the range of the value estimated from systematic review (and assuming that the assumed values were actually the case), the transmissibility at a population level is likely elevated through the increase of people with multiple partners. However, when the value *q* was slightly higher than the empirically estimated range, the reproduction number appeared to decrease with the increased proportion of people with multiple partners. It is striking that we cannot describe the transmission potential in relation to concurrency in a monotonic fashion. Depending on parameters and actual coverage of condom use, it should be remembered that the increase of multiple partners may lead to decreased reproduction number.

The resulting take-home message is straightforward. If a positive association between the reproduction number and the proportion of people with multiple partners is the case, public health interventions should be stressed on sexually high risk population with casual or multiple partners. Nevertheless, if the correlation between condom use and the type or number of sexual partners is actually greater than that we estimated, the abovementioned association may be reversed, and then, it may be more beneficial to target people with steady or single partner alone. In other words, depending on the correlation between condom use and type of partnership or concurrency, theoretically supported type of people to be intervened may likely vary. The present study underscores the need to explore the correlation in a variety of settings, e.g. in a closely related group of people including high schools or Universities, or a setting that focuses on contact between commercial sex workers and males.

Considering that our study rested on a simplistic model, three limitations must be noted. First, our model did not rest on very specific disease in mind. For instance, if we handle man-to-man transmissible STI, we must have accounted for men having sex with men (or homosexual population). We ignored this matter for the simple exposition of our theoretical finding. Second, we did not model and examine the type of partnership (casual and steady partnership) in the mathematical model. Third, whereas we simplified the sexual contact pattern as single/multiple or steady/casual, concurrency and type of partnership might be associated somehow. Sexual contact pattern might have been oversimplified to be immediately applied to concrete examples of STI.

Despite these limitations, we believe that the present study successfully clarified the critical fact that individuals who have multiple partnerships use condom more frequently than individuals who have single relationship alone. To consider possible public health countermeasures against STI, it is advised to explore the correlation between condom use and sexual contact pattern so that the most important target host can be objectively identified.

## Conclusions

Depending on the correlation between condom use and type of partnership or concurrency, increase of people with multiple partners may sometimes result in decrease in the reproduction number, and theoretically supported target host to be intervened may likely vary. The present study underscores the need to explore the correlation in a variety of settings, e.g. in a closely related group of people including high schools or Universities.

## Abbreviations

HIV: Human immunodeficiency virus
HPV: Human papilloma virus
IDU: Intravenous/injection drug users
NGM: Next generation matrix
PID: Pelvic inflammatory disease
STI: Sexually transmitted infection

## References

[1] Deschryver A, Meheus A (1990). Epidemiology of sexually-transmitted diseases – the global picture. Bull World Health Organ.

[2] UNAIDS (2016). Fact Sheet 2016 Global statistics – sexually transmitted infections(STIs).

[3] Haggerty CL, Gottlieb SL, Taylor BD, Low N, Xu F, Ness RB (2010). Risk of Sequelae after chlamydia trachomatis genital infection in women. AJ Infect Dis.

[4] Paavonen J, Eggert-Kruse W (1999). Chlamydia trachomatis: impact on human reproduction. Hum Reprod Update.

[5] Davey DLJ, Shull HI, Billings JD, Wang D, Adachi K, Klausner JD (2016). Prevalence of curable sexually transmitted infections in pregnant women in low- and middle-income countries from 2010 to 2015 a systematic review. Sex Transm Dis.

[6] Fleming DT, Wasserheit JN (1999). From epidemiological synergy to public health policy and practice: the contribution of other sexually transmitted diseases to sexual transmission of HIV infection. Sex Transm Inf.

[7] Davis KR, Weller SC (1999). The effectiveness of condoms in reducing heterosexual transmission of HIV. Fam Plan Perspect.

[8] Bogart LM, Skinner D, Weinhardt LS, Glasman L, Sitzler C, Toefy Y, Kalichman SC (2011). HIV misconceptions associated with condom use among black south Africans: an exploratory study. Afr J AIDS Res.

[9] Macaluso M, Demand MJ, Artz LM, Hook EW (2000). Partner type and condom use. AIDS.

[10] Cochran WG (1954). The combination of estimates from different experiments. Biometrics.

[11] Higgins JPT, Thompson SG (2002). Quantifying heterogeneity in a meta-analysis. Stat Med.

[12] Hethcote HW. Modeling heterogeneous mixing in infectious disease dynamics. In: Models for Infectious Human Diseases. Their Structure and Relation to Data. (Eds) Isham V, Medley G. Cambridge: Cambridge University Press. 1996: pp. 215–238.

[13] Catania JA, Stone V, Binson D, Dolcini MM (1995). Changes in condom use among heterosexuals in wave 3 of the AMEN survey. J Sex Res.

[14] Ellen JM, Adler N, Gurvey JE, Millstein SG, Tschann J (2002). Adolescent condom use and perceptions of risk for sexually transmitted diseases. Sex Transm Dis.

[15] Evans BA, Bond RA, MacRae KD (1997). Sexual relationships, risk behaviour, and condom use in the spread of sexually transmitted infections to heterosexual men. Genitourin Med.

[16] Hargreaves JR, Morison LA, Kim JC, Busza J, Phetla G, Porter JDH, Watts C, Pronyk PM (2009). Characteristics of sexual partnerships, not just of individuals, are associated with condom use and recent HIV infection in rural South Africa. AIDS Care.

[17] Howard MM, Fortenberry JD, Blythe MJ, Zimet GD, Orr DP (1999). Patterns of sexual partnerships among adolescent females. J Adolesc Health.

[18] Meekers D (2003). Patterns of condom use in urban males in Zimbabwe: evidence from 4600 sexual contacts. AIDS Care.

[19] Nq’weshemi JZL, Boerma JT, Pool R, Barongo L, Senkoro K, Maswe M, Isingo R, Schapink D, Nnko S, Borgdorff MW (1996). Changes in male sexual behaviour in response to the AIDS epidemic: evidence from a cohort study in urban Tanzania. AIDS.

[20] Peterman TA, Tian LH, Warner L, Satterwhite CL, Metcalf CA, Malotte KC, Paul SM, Douglas JM, the RESPECT-2 study group (2009). Condom use in the year following a sexually transmitted disease clinic visit. Int J STD AIDS.

[21] Staras SAS, Livingston MD, Maldonado-Molina MM, Komro KA (2013). The influence of sexual partner on condom use among urban adolescents. J Adolesc Health.

[22] Ssewanyana D, Sebena R, Petkeviciene J, Luk ÁCA, Miovsky M, Stock C (2015). Condom use in the context of romantic relationships: a study among university students from 12 universities in four central and eastern European countries. Eur J Contracept Reprod Heal Care.

[23] Turchik JA, Gidycz CA (2012). Exploring the intention-behavior relationship in the prediction of sexual risk behaviors: can it be strengthened?. J Sex Res..

[24] Cushman LF, Romero D, Kalmuss D, Davidson AR, Heartwell S, Rulin M (1998). Condom use among women choosing long-term hormonal contraception. Fam Plan Perspect.

[25] Johnson EH, Gant L, Hinkle YA, Gilbert D, Willis C, Hoopwood T (1992). Do African-American men and women differ in their knowledge about AIDS, attitudes about condoms, and sexual behaviors?. J Natl Med Assoc.

[26] Plichta SB, Weisman CS, Nathanson CA, Ensminger ME, Robinson JC (1992). Partner-specific condom use among adolescent women clients of a family planning clinic. J Adolesc Health.

[27] Hyman JM, Li J (1997). Disease transmission models with biased partnership selection. Appl Num Math.

[28] Kretzschmar M, Heijne JCM (2017). Pair formation models for sexually transmitted infections: a primer. Infect Dis Model.

[29] Turner KM, Adams EJ, Gay N, Ghani AC, Mercer C, Edmunds WJ (2006). Developing a realistic sexual network model of chlamydia transmission in Britain. Theor Biol Med Model.

[30] Althaus CL, Turner KM, Schmid BV, Heijne JC, Kretzschmar M, Low N (2012). Transmission of chlamydia trachomatis through sexual partnerships: a comparison between three individual-based models and empirical data. J R Soc Interface.

[31] MI C, Ghani AC (2010). Populations and partnerships: insights from metapopulation and pair models into the epidemiology of gonorrhoea and other sexually transmitted infections. Sex Transm Infect.

[32] Azizi A, Rios-Soto K, Mubayi A, Hyman JM (2017). A risk-based model for predicting the impact of using condoms on the spread of sexually transmitted infections. Infect Dis Model.

[33] Nishiura H (2010). Correcting the actual reproduction number: a simple method to estimate R0 from early epidemic growth data. Int J Environ Res Public Health.

